# Endothelial Connexin37 and Connexin40 participate in basal but not agonist-induced NO release

**DOI:** 10.1186/s12964-015-0110-1

**Published:** 2015-07-22

**Authors:** Merlijn J Meens, Florian Alonso, Loïc Le Gal, Brenda R Kwak, Jacques-Antoine Haefliger

**Affiliations:** Department of Pathology and Immunology, University of Geneva, 6th floor, 1 Rue Michel-Servet, 1211 Geneva, Switzerland; Department of Medical Specialties - Cardiology, University of Geneva, Geneva, Switzerland; Department of Medicine, University Hospital, CHUV, Lausanne, Switzerland

**Keywords:** Connexin, Nitric oxide, Endothelium

## Abstract

**Background:**

Connexin37 (Cx37) and Cx40 are crucial for endothelial cell-cell communication and homeostasis. Both connexins interact with endothelial nitric oxide synthase (eNOS). The exact contribution of these interactions to the regulation of vascular tone is unknown.

**Results:**

Cx37 and Cx40 were expressed in close proximity to eNOS at cell-cell interfaces of mouse aortic endothelial cells. Absence of Cx37 did not affect expression of Cx40 and a 50 % reduction of Cx40 in Cx40^+/−^ aortas did not affect the expression of Cx37. However, absence of Cx40 was associated with reduced expression of Cx37. Basal NO release and the sensitivity for ACh were decreased in Cx37^−/−^ and Cx40^−/−^ aortas but not in Cx40^+/−^ aortas. Moreover, ACh-induced release of constricting cyclooxygenase products was present in WT, Cx40^−/−^ and Cx40^+/−^ aortas but not in Cx37^−/−^ aortas. Finally, agonist-induced NO-dependent relaxations and the sensitivity for exogenous NO were not affected by genotype.

**Conclusions:**

Cx37 is more markedly involved in basal NO release, release of cyclooxygenase products and the regulation of the sensitivity for ACh as compared to Cx40.

**Electronic supplementary material:**

The online version of this article (doi:10.1186/s12964-015-0110-1) contains supplementary material, which is available to authorized users.

## Background

Connexins form a family of transmembrane proteins [1, 2]. They are inserted into cell membranes as hexamers called connexons. Gap junction channels, structures that arise after docking of two connexons from neighbouring cells, allow diffusion of ions and small metabolites (e.g. ATP, Ca^2+^ and cAMP) between cells and are thus implicated in direct cell-to-cell communication and synchronization of tissue responses [3]. Under specific conditions, connexons may also function as hemichannels allowing for diffusion of ions and small metabolites from the cytosol to the extracellular space or *vice versa* [4].

It is now increasingly recognized that connexins do not only function as connexons or gap junction channels; they can also regulate the function of other proteins via protein-protein interactions [5–7]. It has for instance been demonstrated that Cx37 interacts with eNOS [8]. Moreover, these interactions between Cx37 and eNOS reduce basal nitric oxide (NO) release *in vitro* [8]. However, a specific role for Cx37-eNOS interactions at the organ level remains to be addressed. In addition, it has been shown that Cx40 interacts with eNOS and that Cx40-deficient mice are characterized by reduced endothelium-dependent NO-mediated relaxations [9]. However, Cx37 is also reduced in Cx40-deficient mice [9]. It is thus unclear whether Cx37-eNOS interactions, Cx40-eNOS interactions or both are responsible for the vascular phenotype of Cx40-deficient mice. Therefore, the current study addressed whether Cx37-deficiency, Cx40-deficiency or a 50 % reduced expression of Cx40 affects basal or agonist-induced release of NO. The key findings are that mainly Cx37 directly modulates i) the spontaneous release of NO from mouse aortic endothelium, ii) the sensitivity of mouse aortic endothelial cells for ACh and iii) the agonist-induced release of endothelium-derived COX-generated contractile factors.

## Results and discussion

### Mouse aortic endothelial cells express Cx37 and Cx40 but not Cx43s

**Fig. 1:**
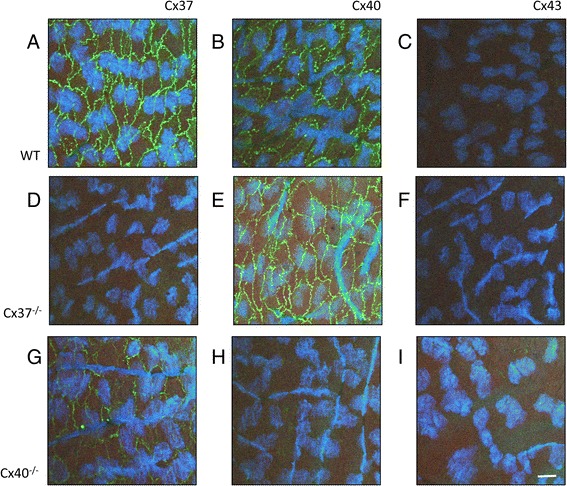
Mouse aortic endothelial cells express Cx37 and Cx40 at cell-cell interfaces. **a**-**c** Representative images of *en face* confocal immunofluorescent stainings for Cx37 (**a**), Cx40 (**b**) or Cx43 (**c**) in wild-type (WT) mouse aortic endothelium, respectively. **d**-**f** Representative images of *en face* confocal immunofluorescent stainings for Cx37 (**d**), Cx40 (**e**) or Cx43 (**f**) in Cx37^−/−^ mouse aortic endothelium, respectively. **g**-**i** Representative images of *en face* confocal immunofluorescent stainings for Cx37 (**g**), Cx40 (**h**) or Cx43 (**i**) in Cx40^−/−^ mouse aortic endothelium, respectively. Scalebar equals 15 μM

The cellular localization of Cx37, Cx40 and Cx43 was studied by immunofluorescence performed on *en face* preparations of WT, Cx37^−/−^ and Cx40^−/−^ aortas. These experiments showed that WT mouse aortic endothelial cells expressed Cx37 and Cx40 at cell-cell interfaces whereas Cx43 was barely detectable (Fig. 1a-c). In Cx37^−/−^ endothelium, Cx37 and Cx43 were not detected and the immunosignal for Cx40 was comparable to the Cx40 immunosignal in WT endothelium (Fig. 1d-f). Finally, in Cx40^−/−^ endothelium, Cx40 and Cx43 were not found and the immunosignal for Cx37 was reduced as compared to the Cx37 immunosignal in WT endothelium (Fig. 1g-i). In summary, i) Cx37 and Cx40 are expressed at intercellular junctions of mouse aortic endothelial cells, ii) the level of Cx37 expression seems to be dependent on the expression of Cx40 and iii) Cx43 is barely detectable in mouse aortic endothelium. Expression of Cx43 in rat aortic endothelium is mainly restricted to areas exposed to disturbed blood flow [10], hence a low level of Cx43 expression in mouse aortic endothelial cells of the thoracic aorta was in line with expectations. Moreover, this study was performed on a part of the thoracic aorta that is exposed to high laminar shear stress, a condition that likely increases expression of Cx37 expression due to its effect on the transcription factor KLF2 [11] and that might increase expression of Cx40 due to the activation of Akt similar to the situation in arterioles [12]. Interestingly, the immunosignal for Cx37 seemed reduced in Cx40^−/−^ mouse aortic endothelial cells whereas the immunosignal for Cx40 was not altered in Cx37^−/−^ endothelium. Thus, there might be interdependence of Cx37 and Cx40 expression.

### Cx40-deficiency affects endothelial Cx37 expression

**Fig. 2:**
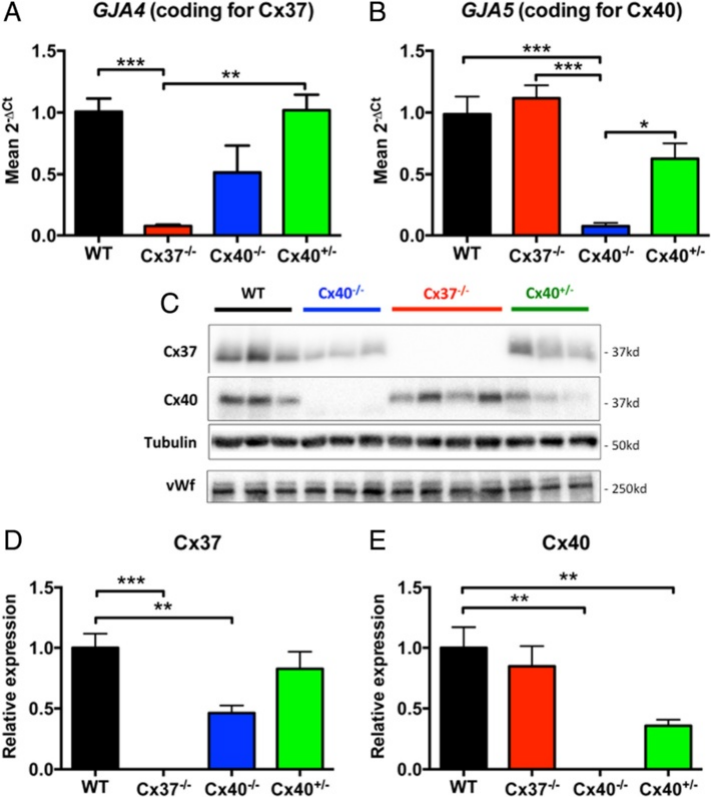
Cx37-deficiency does not affect expression of Cx40 while Cx40-deficiency reduces expression of Cx37. **a**-**b** Total mRNA was isolated from aortas obtained from WT, Cx37^−/−^, Cx40^−/−^ or Cx40^+/−^ mice (*N* = 3). Thereafter, the expression of *Gja4* (A, the gene coding for Cx37) or *Gja5* (B, the gene coding for Cx40) was assessed by real-time PCR. Cx40 is expressed at WT levels in the Cx37^−/−^ aortas while Cx37 showed large variation and tended to be reduced in the Cx40^−/−^ aortas (*p* = 0.08). Moreover, expression of Cx37 is not reduced in Cx40^+/−^ aortas. **c** Representative image of Cx37 and Cx40 protein expression in scraped mouse aortic endothelial cells as assessed by western blot. Tubulin and von Willebrand factor (vWf) were assessed for equal protein loading. **d** Cx37 protein quantification (*N* = 6–8). Similar to the expression of Cx37 at the level of RNA, Cx37 expression was reduced by half in Cx40^−/−^ aortic endothelial cells whereas Cx37 expression was not significantly reduced in Cx40^+/−^ endothelium. **e** Cx40 protein quantification (*N* = 6–8). Cx40 was diminished by half in Cx40^+/−^ endothelial cells and was not altered in Cx37^−/−^ endothelium. Values are expressed as mean ± SEM. *, ** or *** *p* < 0.05, 0.01 or 0.001 *vs* WT control, respectively

To further study whether expression of Cx37 affects the expression of Cx40 or *vice versa,* the expression of *Gja4* (the gene coding for mouse Cx37) or *Gja5* (the gene coding for mouse Cx40) was quantified by real-time PCR performed on total aortic mRNA. As expected, mRNA coding for Cx37 was detectable in WT aortas but not in Cx37^−/−^ aortas (Fig. 2a). Moreover, mRNA coding for Cx37 showed a large variation and tended to be reduced in Cx40^−/−^ aortas (*p* = 0.08) and was comparable to WT levels in Cx40^+/−^ aortas (Fig. 2a). These findings are in line with an earlier study showing that endothelial Cx40-deficient mice do not display significantly reduced Cx37 mRNA levels in both the aorta and the cremaster muscle [13]. mRNA coding for Cx40 was expressed at similar levels in WT and Cx37^−/−^ aortas (Fig. 2b). mRNA expression does not necessarily predict protein expression due to for example posttranslational modifications, alternative splicing, etc. [14]. Expression of Cx37 and Cx40 protein content was therefore also assessed by western blotting performed on mouse aortic endothelial cells collected from WT, Cx37^−/−^ or Cx40^−/−^ mice (see Fig. 2c for a representative example from 6 to 8 mice). These experiments confirmed that Cx37 was reduced in Cx40^−/−^ aortic endothelial cells as compared to WT endothelium (Fig. 2d, e). Thus, in line with earlier findings in mouse arterioles [15] and the mouse aorta [9, 16], Cx37 was reduced in Cx40^−/−^ endothelial cells. Moreover, in line with yet another study [17], Cx40 expression was not affected by absence of Cx37. Subsequently, the expression of Cx37 and Cx40 was studied in Cx40^+/−^ mouse aortic endothelial cells. As expected, these studies showed that the expression of Cx40 tended to be reduced by about 50 % in Cx40^+/−^ endothelial cells at the level of mRNA (Fig. 2b; *p* = 0.09) and was significantly reduced at the level of protein (Fig. 2c and e; *p* < 0.01). However, the expression of Cx37 in Cx40^+/−^ aortic endothelial cells was not significantly different from its expression in WT aortic endothelium (Fig. 2a, c and d). In conclusion, expression of Cx40 seems to be necessary for proper expression of Cx37 in mouse aortic endothelium. However, even low levels of Cx40 expression, like in Cx40^+/−^ mice, are sufficient to maintain Cx37 expression at a level comparable to WT aortic endothelium. As recently demonstrated [13], maintenance of endothelial Cx37 may depend on the presence of Cx40 rather than to its channel function. Altogether, by using Cx40^+/−^ aortas the effect of a specific reduction in Cx40 expression on vasomotor function can be studied independent from a reduction in Cx37.

### Cx37 and Cx40 are in close proximity to eNOS in mouse aortic endothelium

**Fig. 3:**
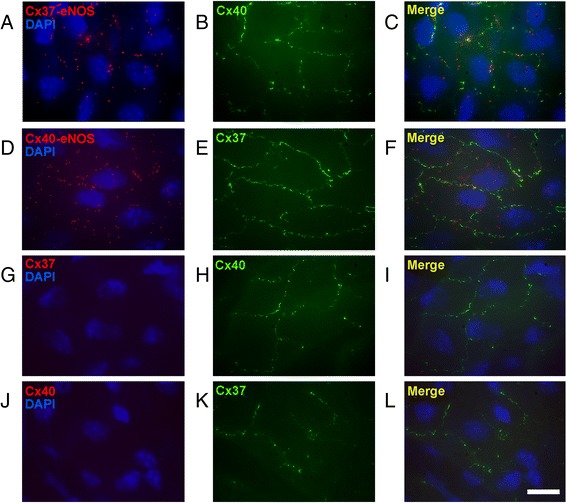
Interactions between connexins and eNOS are present at cell-cell interfaces between mouse aortic endothelial cells. Representative images of proximity ligation assays performed with antibodies targeting Cx37 and eNOS or Cx40 and eNOS. **a** Close proximity of Cx37 and eNOS in WT mouse aortic endothelium. **b** Cx40 staining to highlight the intercellular gap junctions. **c** Merge of (**a**) and (**b**) highlighting close proximity of eNOS and Cx37 at the gap junctions between endothelial cells. **d** Close proximity of Cx40 and eNOS in WT endothelium. **e** Cx37 staining to highlight the intercellular gap junctions. **f** Merge of (**d**) and (**e**) highlighting close proximity of eNOS and Cx40 at the gap junctions between endothelial cells. **g**-**i** Control assays revealed that the intense red staining observed in **a**-**c** was no longer observed after omitting the eNOS antibody from the proximity ligation assays (**g**-**i**) even though cell-cell junctions could clearly be visualized (**h**-**i**). **j**-**l**: Similar control stainings, which revealed similar results, were performed with the Cx40 antibodies, but not the Cx37 antibodies present during the proximity ligation assays. Scalebar equals 20 μM

Similar to Cx37 and Cx40, eNOS can also be found at cell-cell interfaces in aortic endothelium. Moreover, Cx37 and Cx40 have been suggested to interact with eNOS in cellular junctions of endothelial cells [8, 9]. Here, the possible interactions between Cx37, Cx40 and eNOS were studied using *in situ* proximity ligation assays. Cx37 and eNOS (Fig. 3a-c), and Cx40 and eNOS (Fig. 3d-f) were detected in close proximity at cell-cell interfaces in mouse aortic endothelium. Importantly, no signal was detected in negative controls from which the antibody detecting eNOS was omitted (Fig. 3g-i and j-l). Moreover, interactions between Cx37 and Cx40 were detected at intercellular junctions of WT aortic endothelial cells (Additional file 1: Figure S1 A-B) and these were absent between Cx40^−/−^ endothelial cells (Additional file 1: Figure S1 C-D), further illustrating the specificity of the *in situ* proximity ligation assays. In conclusion, both Cx37 and Cx40 are in close proximity with eNOS at intercellular junctions of mouse aortic endothelial cells. Hence, both Cx37 and Cx40 might regulate eNOS-mediated aspects of vasomotor function.

### Cx37 is involved in basal NO-mediated vasomotor responses

**Fig. 4:**
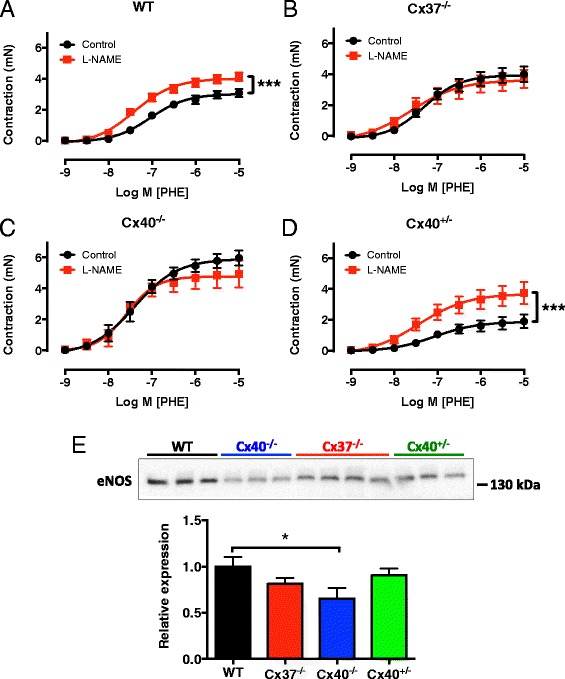
Cx37 is implicated in basal NO release. Aortic segments obtained from WT, Cx37^−/−^, Cx40^−/−^ or Cx40^+/−^ mice were mounted in a wire-myograph and concentration-response curves for PHE were generated in presence or absence of L-NAME (100 μM). **a** Inhibition of NO synthesis by L-NAME increased the contractile amplitude for PHE in WT aortas indicating presence of basal NO release. This effect of L-NAME was not observed in aortas isolated from Cx37^−/−^ (**b**) or Cx40^−/−^ (**c**) mice. In contrast (**d**), in aortas obtained from Cx40^+/−^ mice L-NAME did increase the contractile amplitude of PHE. Values are expressed as mean ± SEM. *N* = 6–10. **, *** *p* < 0.01, 0.001 *vs* control, respectively. **e** Representative image and quantification of eNOS protein expression in protein samples from scraped mouse aortic endothelial cells as assessed by western blot. Values are expressed as mean ± SEM. *N* = 6–8. * *p* < 0.05 *vs* WT

**Fig. 5:**
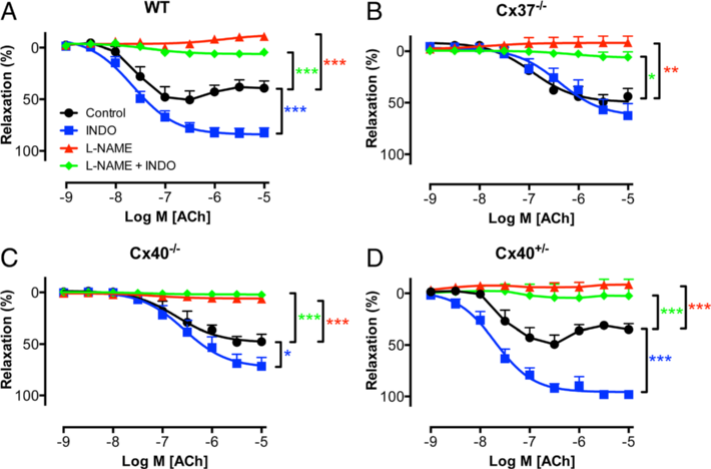
Cx37 may be implicated in the release of COX products but not in activated NO release. Concentration-response curves for the endothelium-specific agonist ACh were generated in PHE pre-contracted aortas obtained from WT, Cx37^−/−^, Cx40^−/−^ or Cx40^+/−^ mice. **a** WT aortas displayed relaxations in response to ACh (*black*) which were i) increased in presence of INDO (*blue*) and ii) inhibited in presence of L-NAME (*red*) or in presence of L-NAME and INDO (*green*). **b** Cx37^−/−^ aortas displayed relaxation responses to ACh (*black*) which were i) unaffected by presence of INDO (*blue*), ii) inhibited in presence of L-NAME (*red*) or in presence of L-NAME and INDO (*green*). **c** Aortas obtained from Cx40^−/−^ mice (**c**) displayed relaxations in response to ACh (*black*) which were i) slightly, but significantly, increased in presence of INDO (*blue*) and ii) inhibited in presence of L-NAME (*red*) or in presence of L-NAME and INDO (*green*). **d** Aortas obtained from Cx40^+/−^ mice displayed relaxations in response to ACh (*black*) which were i) increased in presence of INDO (*blue*) and ii) inhibited in presence of L-NAME (*red*) or in presence of L-NAME and INDO (*green*). Values are expressed as mean ± SEM. *N* = 6–10. *, ** or *** *p* < 0.05, 0.01 or 0.001 *vs*. control, respectively

The data in this study show that Cx37 and Cx40 are i) expressed at cell-cell interfaces and ii) in a close proximity to eNOS. Thus, they suggest that endothelial Cx37 expression may be dependent on proper Cx40 expression. Moreover, these findings imply that Cx37 and/or Cx40 could regulate eNOS-dependent NO production *in vivo* and warrant studies towards a possible role of interactions between Cx37 or Cx40 and eNOS in basal NO release at the organ level. Consequently, we assessed phenylephrine (PHE)-induced contractions in aortas isolated from WT, Cx37^−/−^, Cx40^−/−^ and Cx40^+/−^ mice using wire-myography. Initially, PHE-induced in absence of pharmacological inhibitors were studied. These experiments showed that the amplitude of the PHE-induced contractions was significantly enhanced in Cx37^−/−^ and Cx40^−/−^ aortas as compared to WT aortas (Fig. 4, Table 1). The amplitude of these contractions, which result from activation of adrenergic receptors expressed by vascular smooth muscle cells, depends on i) the balance between the relative amount of vasoconstrictors and vasodilators present in the organ bath and ii) the amount of vascular smooth muscle cells in the vessel wall. Indeed, as expected, given the hypertension-induced hypertrophy of the vascular smooth muscle in the Cx40^−/−^ aortas [9], the PHE-induced contractions were significantly enhanced in the Cx40^−/−^ aortas as compared to the Cx37^−/−^ and WT aortas (Fig. 4, Table 1). Interestingly, in case of basal NO release, one would expect that presence of the NOS inhibitor *N*^ω^-nitro-l-arginine methyl ester (L-NAME) would increase the contractile amplitude. Indeed, the amplitude of PHE-induced contractions was significantly increased in aortas isolated from WT mice in presence of L-NAME (Fig. 4a, Table 1). This was not the case in aortas isolated from Cx37^−/−^ (Fig. 4b, Table 1) or Cx40^−/−^ mice (Fig. 4c, Table 1). Presence of L-NAME significantly increases PHE-induced contractions in aortas isolated from Cx40^+/−^ mice that are characterized by a 50 % reduced expression of Cx40 but normal Cx37 expression levels (Fig. 4d, Table 1). In conclusion, our observations in Cx37^−/−^, Cx40^+/−^ and Cx40^−/−^ aortas show that Cx37 is implicated in basal NO release. Although it cannot be completely ruled out that the remaining level of Cx40 expression in Cx40^+/−^ aortas is sufficient for the generation of basal NO levels comparable to WT aortas, the apparent involvement of Cx40 is most likely explained by an indirect mechanism (e.g. via the regulation of Cx37 expression). However, Cx37 seems to be necessary for a direct regulation of eNOS function since basal NO release in Cx37^−/−^ aortas, which have normal Cx40 expression levels, was reduced. Exactly how Cx37 regulates the basal release of NO is currently unclear. One possibility would be via direct effects on eNOS expression levels. We therefore studied eNOS expression by western blotting of mouse aortic endothelial cell lysates collected from WT, Cx37^−/−^, Cx40^−/−^ or Cx40^+/−^ mice. In line with previous findings [9, 18], eNOS expression was reduced in Cx40^−/−^ endothelium, but expression levels were similar in Cx37^−/−^, Cx40^+/−^ and WT mice (Fig. 5e). NO production in the endothelium results from a complex series of interactions between eNOS, Ca^2+^, calmodulin, caveolin and tetrahydropiopterin [19]. Theoretically, Cx37 could alter e.g. binding of any of these factors to eNOS thereby changing its activity and increasing NO release. Indeed, the C-terminal tail of Cx37 interacts with eNOS at its reductase domain, a part that also contains a caveolin-1 binding site [8]. Finally, one should keep in mind that “mixed channels” composed of both Cx37 and Cx40 may be involved in regulation of NO release as well.

**Table 1:** Characteristics of PHE-induced contractions in presence or absence of L-NAME

	E_max_ (mean (mN) ± SEM)				EC_50_ (mean (nM) 95 % CI)			
	WT	Cx37^−/−^	Cx40^−/−^	Cx40^+/−^	WT	Cx37^−/−^	Cx40^−/−^	Cx40^+/−^
Control	3.1 ± 0.2	4.0 ± 0.5 $$$, §§§	6.0 ± 0.5 $$$	1.9 ± 0.4 §§§	91 95 % CI [57, 145]	48 95 % CI [25, 94]	39 95 % CI [20, 78]	84 95 % CI [24, 291]
L-NAME	4.1 ± 0.3***	3.7 ± 0.6	4.9 ± 0.9	3.7 ± 0.7***	39 95 % CI [24, 62]	24 95 % CI [5, 115]	26 95 % CI [12, 57]	37 95 % CI [8, 160]

*N* = 6–10. ***, $$$, §§§ *p* < 0.001 *vs* the respective control, WT or Cx40^−/−^

### Involvement of Cx37 or Cx40 in agonist-induced NO release

Basal and activated NO release do not necessarily depend on similar mechanisms. Findings regarding basal NO release can therefore not always be extrapolated to activated NO release. For that reason, CRCs to acetylcholine (ACh) during PHE-induced pre-contractions in aortas isolated from WT, Cx37^−/−^, Cx40^−/−^ or Cx40^+/−^ mice were studied. In WT aortas ACh induced relaxations that reached maximal amplitudes at an ACh concentration of 300 nM (Fig. 5a, Table 2). However, in presence of indomethacin (INDO) the CRCs for ACh only reached maximal amplitude at an ACh-concentration of 10 μM (Fig. 5a, Table 2). Finally, ACh-induced relaxations were dramatically reduced in presence of L-NAME (maximal relaxation −11 ± 2 %) and in presence of L-NAME + INDO (Fig. 5a; maximal relaxation 4 ± 2 %). Thus, in WT aortas ACh-induced relaxations are fully mediated by NO and can be partially masked by INDO-sensitive contractile factors at high ACh concentrations. Surprisingly, the CRCs for ACh that were generated in Cx37^−/−^ aortas were characterized by a lower sensitivity compared to CRCs generated in WT aortas (Table 3) and were unaffected by INDO (Fig. 5b, Table 2). Similar to its effect in WT-aortas, presence of L-NAME drastically reduced the effect of ACh in Cx37^−/−^ aortas (Fig. 5b). CRCs for ACh generated in aortas from Cx40-deficient animals were also characterized by a lower sensitivity compared to CRCs generated in WT aortas (Table 3). However, INDO displayed a slight but significant effect on the maximal effect of ACh in Cx40^−/−^ aortas (Fig. 5c, Table 2). In line with expectations, L-NAME also reduced the effects of ACh in the Cx40^−/−^ aortas (Fig. 5c, Table 2). Finally, CRCs for ACh generated in Cx40^+/−^ aortas were similar in all aspects to those generated in WT aortas (Fig. 5d, Table 2 and Table 3). In summary, Cx37 and Cx40 do probably not affect the activated NO release. Cx37, however, seems implicated in the release of constricting COX products in response to higher ACh concentrations since a significant effect on INDO was not observed in Cx37^−/−^ aortas (which express Cx40 at WT levels) but was observed in Cx40^−/−^ aortas (which express low levels of Cx37) and in Cx40^+/−^ aortas (which express Cx37 at WT levels). Furthermore, changes in the expression of Cx37 are most likely also responsible for the changes in the sensitivity for ACh that were observed since these changes were fully normalized in Cx40^+/−^ aortas. However, half of Cx40 expression may also be sufficient for normal endothelial function. Surprisingly, ACh-induced NO-mediated dilatation was not affected by Cx40 (or by Cx37; this study) in contrast to earlier work from our group [9]. The reasons for this discrepancy are not entirely clear but are most likely due to methodological differences between the studies (e.g. the use of different agonists to induce precontractions, cumulative *vs* non-cumulative CRCs, different timing of agonist application).

**Table 2:** E_max_ of ACh-induced relaxations during PHE-induced contractions in presence or absence of INDO, L-NAME or a combination of the former

	E_max_ (mean (% relaxation) ± SEM)			
	WT	Cx37^−/−^	Cx40^−/−^	Cx40^+/−^
Control	51 ± 9	50 ± 7	47 ± 7	50 ± 9
INDO	83 ± 5***	63 ± 12	71 ± 8*	98 ± 3**
L-NAME	−11 ± 2***	−8 ± 6***	6 ± 4***	−8 ± 4***
L-NAME + INDO	4 ± 2***	6 ± 5***	2 ± 3***	3 ± 16**

*N* = 6–10. *, ** or *** *p* < 0.05, 0.01 or 0.001 *vs* control

**Table 3:** EC_50_ (mean (nM) 95 % CI) for ACh during PHE-induced contraction in presence of INDO

WT	Cx37^−/−^	Cx40^−/−^	Cx40^+/−^
23 95 % CI [15, 35]	409 95 % CI [153, 1088]*	274 95 % CI [125, 604]*	19 95 % CI [11, 32]

* *p* < 0.05 *vs* WT

### Involvement of Cx37 or Cx40 in release of contractile factors

Various studies have described endothelium-dependent contractile effects of high ACh concentrations [20–23]. Indeed, in the current study the lower ACh concentrations induced relaxations that no longer increased at higher ACh concentrations in WT aortas. It has been shown that COX1-derived products mediate contractile effects of ACh in the mouse aorta [20]. We therefore assessed the effect of blocking cyclooxygenases using INDO (a dual COX1/COX2 inhibitor) on ACh-induced relaxations. Indeed, presence of INDO significantly increased the maximal effect of ACh in WT, Cx40^−/−^ and Cx40^+/−^ but not Cx37^−/−^ aortas. Interestingly, it has been suggested that gap junctional communication between endothelial cells or, alternatively, between endothelium and smooth muscle cells could be responsible for the contractile effects of ACh in the aorta of spontaneously-hypertensive rats [21]. However, this latter study relied on rather non-specific chemical inhibitors such as carbenoxolone and only addressed the role of connexins in the context of hypertension, a disease that is well-known to alter the distribution of connexins in the vascular wall [24]. As effects of INDO on the Emax of ACh-induced relaxations was observed in WT, Cx40^+/−^ and Cx40^−/−^ aortas but not in Cx37^−/−^ aortas, we conclude that Cx37 is necessary to allow the effect of INDO.

### Connexin deficiency does not alter the sensitivity for NO

The altered sensitivity for ACh that was observed in Cx37^−/−^ and Cx40^−/−^ aortas may be due to i) a changed sensitivity of the aortic endothelial cells for ACh, ii) an altered eNOS expression level (in the Cx40^−/−^ but not the Cx37^−/−^ aortas) or iii) a changed sensitivity of the vascular smooth muscle cells for NO. The latter possibility was tested by generating CRCs to an NO donor (sodium-nitroprusside, (SNP)) during PHE-induced precontractions (in presence of L-NAME and INDO to avoid effects of endogenous NO or COX-products, respectively) in WT, Cx37^−/−^, Cx40^−/−^ or Cx40^+/−^ aortas. These experiments showed that aortas isolated from WT, Cx37^−/−^, Cx40^−/−^ or Cx40^+/−^ mice were equally sensitive to NO (Table 4). In summary, the changed sensitivity for ACh, which was observed in Cx37^−/−^ and Cx40^−/−^ aortas, was not due to alterations at the smooth muscle level (e.g. a decreased sensitivity of the ‘NO receptor’ soluble guanylyl cyclase (sGC)) but likely reflected endothelial changes. Indeed, involvement of Cx40 (and most likely Cx37) in the endothelial Ca^2+^ agonist responses, which govern NO release, are agonist specific [25]. *I.e.* ATP-induced increases in intracellular Ca^2+^ in mouse endothelium are homogeneous and do not require synchronization by connexins [25]. In contrast, Ca^2+^ elevations mediated by ACh are heterogenous and require synchronization by connexins to reach other cells [25]. This observation sparks thoughts such as ‘thus, the diminished response to ACh makes sense since ACh-induced responses require synchronization via connexins’. However, although this is theory true for the total amount of NO released by aortic endothelial cells exposed to a certain ACh concentration (an effect that was not actually observed in this study), this theory does not hold regarding sensitivity changes. Such changes most likely involve conformational changes of the M3-receptor or enzymes, like eNOS, functioning downstream of M3-receptor activation but upstream of the activation of sGC. eNOS seems a likely candidate since Cx37 and Cx40 have not been shown to interact with M3 receptors (nor do they co-localize with M3 receptors in mouse aortic endothelial cells (see Fig. 4 in [25])) but do interact with this NO-generating enzyme. Tantalizingly, if the basal intracellular Ca^2+^ concentration is unaffected by genotype then a decreased eNOS sensitivity for Ca^2+^ could explain both the effect observed regarding basal NO release (decreased NO released at fixed Ca^2+^ concentration) and the apparent effect on sensitivity of ACh (increasing concentrations of ACh result in increasing concentrations of intracellular Ca^2+^ that do not have similar effects due to the altered Ca^2+^ sensitivity of eNOS). Obviously, this will be the topic of further studies.

**Table 4:** EC_50_ (mean (nM) 95 % CI) for SNP during PHE-induced contraction in presence of L-NAME and INDO

WT	Cx37^−/−^	Cx40^−/−^	Cx40^+/−^
7 95 % CI [4, 14]	5 95 % CI [2, 10]	3 95 % CI [1, 30]	4 95 % CI [1, 20]

## Conclusions

In summary, the current study was conducted to address whether Cx37 or Cx40 is more pronouncedly involved in the regulation of vasomotor function. The data indicate that Cx37 is more markedly involved in basal NO release, release of COX-products and the regulation of the sensitivity for ACh as compared to Cx40. These findings are exciting since they, for the first time, allow for a discrimination of the functional role of Cx37 and Cx40 in mouse aortic endothelium. However, various questions remain e.g. ‘How does Cx37 modulate basal NO release without modulating eNOS expression levels?’, and importantly, ‘Why is the sensitivity for ACh altered in Cx37^−/−^ aortas’. Clearly, these questions warrant a specific exploration in further studies.

## Methods

The Swiss Federal Veterinary Office approved animal housing and animal experiments. Wild-type (WT, *n* = 10), Cx37^−/−^ (*n* = 10), Cx40^−/−^ (*n* = 8) and Cx40^+/−^ (*n* = 8) mice were progeny of the original colony on a C57BL/6 J background and were housed in a 12-hours day-night cycle [26, 27]. Genotyping was performed as previously described [17, 9].

### Immunofluorescence and *in situ* proximity ligation assays

Immunostainings and *in situ* proximity ligation assays were performed as previously described [9, 11]. In brief, for immunostainings, opened thoracic aortas were fixed in ice-cold methanol, permeabilized with 0.2 % TritonX-100 and blocked in 2 % bovine serum albumin. Primary antibodies (polyclonal rabbit anti-Cx37 (alpha Diagnostic), anti-Cx40 (Chemicon), anti-Cx43 (alpha Diagnostic) were incubated overnight. An Alexa Fluor 488 fluorochrome-conjugated goat-anti rabbit was used for signal detection. Nucleic acids were stained with DAPI and the cytosol was visualized using Evans Blue. Finally, sections were mounted and analysed using a LSM 510 Meta confocal microscope (Zeiss). For the proximity ligation assays, longitudinally opened aortas were fixed in 100 % ethanol, and aortic strips prepared for the simultaneous incubation with rabbit polyclonal antibody against Cx40 (Chemicon) or Cx37 (Biotrend Chemikalien) and mouse monoclonal antibody against eNOS (BD Bioscience). The incubation solution was then supplemented with oligonucleotide-conjugated secondary antibodies (diluted 1:5) and a ligation solution, consisting of two nucleotides and a ligase, to form a circular DNA when eNOS and Cx40 or Cx37 were in close proximity. The circular DNA was then amplified into a long, single-stranded concatemer by rolling circle amplification, using as primer an arm of one of the oligonucleotide-conjugated antibodies. The amplification product was collapsed into a DNA bundle, and detected by hybridizing fluorophore-labeled oligonucleotides (Duolink *in situ* detection reagent red) to the repeated sequences of the amplification product. Under a fluorescence microscope, the result was detected as red spots, which were localized by staining the EC nuclei with DAPI. For the proximity ligation assays performed with antibodies targeting Cx40 and Cx37 in the en face aortic strips, a monoclonal antibody specific to mouse Cx40 was used (Invitrogen).

### Quantitative PCR

RNA isolation, DNAse treatment and reverse transcriptase reactions were performed on whole mouse aortas as previously described [11]. Equal loading was ensured by assessing expression of 18S ribosomal RNA and was similar in all samples. The relative amount of mRNA of interest was calculated after normalization to CD31 for the amount of endothelium in the samples.

### Western blotting

The expression of Cx37 and Cx40 was determined as previously described [9]. In brief, thoracic aortas were longitudinally opened in 100 μl PBS, and were subsequently pinned on a silicone plate with the endothelial-side facing upwards. A scalpel blade was then used to gently scrape off the ECs. ECs in PBS were then centrifuged at 14,000 rpm for 5 min at 4 °C and the pellet was homogenized in lysis buffer (62.5 mM Tris HCl, pH6.8, 5 % SDS, 10 mM EDTA, pH8). Protein content was measured using a detergent-compatible DC protein assay kit (Bio-Rad Laboratories). Samples (20 μg) were equally loaded on a 10 % polyacrylamide gel, separated by electrophoresis and transferred onto PVDF membranes (Immobilon-P; Millipore). Membranes were incubated for 1 h in PBS or TBS containing 5 % milk and 0.1 % Tween 20 (blocking buffer). The membranes were then incubated overnight at 4 °C with one of the following primary antibodies: rabbit polyclonal antibodies against Cx40 (Chemicon), Cx37 (Biotrend Chemikalien) or von Willebrand factor (vWF) (Dako) and mouse monoclonal antibodies against α-tubulin (Sigma-Aldrich) or eNOS (BD Bioscience). The secondary antibodies were horseradish peroxidase-conjugated goat anti-mouse immunoglobulin (Jackson Immuno research); or goat anti-rabbit immunoglobulin (Thermo Scientific), respectively. Bands were developed using enhanced chemiluminescence (Millipore), and visualized using a supercooled CCD camera (Chemidoc XRS). Densitometric analysis was performed using ImageLab Software (3.0.1 Bio-Rad Laboratories). Equal loading was ensured by assessing expression of tubulin and was similar in all the samples. The relative amount of the proteins of interest was calculated after normalization to vWf for the amount of endothelium in the samples.

### Solutions and drugs

Krebs-Ringer bicarbonate-buffered physiological salt buffer (KRB-buffer) contained 118.5 mM NaCl, 4.7 mM KCl, 2.5 mM CaCl_2_, 1.2 mM MgSO_4_, 1.2 mM KH_2_PO_4_, 25.0 mM NaHCO_3_ and 5.5 mM glucose. High K^+^-KRB solution was prepared by replacing NaCl with KCl. Buffers containing intermediate K^+^ concentrations were prepared by mixing volumes of KRB and K^+^-KRB. Acetylcholine (ACh), phenylephrine (PHE), *N*^ω^-nitro-l-arginine methyl ester (L-NAME), sodium-nitroprusside (SNP) and indomethacin (INDO) were obtained from Sigma-Aldrich (Germany). All compounds were dissolved in KRB-buffer with the exception of INDO, which was dissolved in ethanol.

### Wire-myography

Mice were sedated (ketamine (100 mg/kg) + xylazine (100 mg/kg), i.p. injection). Subsequently, the left ventricle was punctured with a 23 G needle. Then, the animals were perfused during 3 min under constant flow (flow rate 3.33 mL/min) with a total volume of 10 mL KRB-buffer (pre-heated at 37 °C). After perfusion, aortic segments were isolated and mounted in a multi-myograph (610 M; DMT, Aarhus, Denmark). After mounting, the segments were stretched to the diameter at which maximal contractile responses to 40 mM K^+^ (K_max_) were obtained (Table 5). Next, to test endothelial integrity, arteries were washed precontracted using PHE (10 μM) and subsequently exposed to ACh (10 μM). Within each genotype, all segments displayed relaxing responses of equal magnitude in response to this muscarinic receptor agonist (data not shown). Thereafter, a concentration-response curve (CRC) for PHE (1 nM – 10 μM) was generated. Subsequently, during the stabilized contractile response to 10 μM PHE, a CRC for ACh (1 nM – 10 μM) was constructed. Twenty minutes after the CRC for ACh the arterial segments were again precontracted using PHE (10 μM). Then, in order to test the NO sensitivity of the vascular smooth muscle cells in the preparation, a CRC for SNP (0.1 nM – 10 μM) was generated during the stabilized contractile response to PHE. These experiments were conducted in parallel in presence of L-NAME (100 μM; to block the synthesis of NO), INDO (10 μM; to block the synthesis of prostaglandins) or a combination of the former.

**Table 5:** Contraction (mN) upon exposure to K^+^ (40 mM) before exposure to pharmacological inhibitors

WT	Cx37^−/−^	Cx40^−/−^	Cx40^+/−^
1.8 ± 0.2	2.8 ± 0.3*	3.9 ± 0.3***	1.8 ± 0.2

*N* = 6–10. * or *** *p* < 0.05 or 0.001 *vs* WT

### Statistical analyses

Results are shown as mean ± SEM. One or two-way ANOVAs followed by Bonferroni’s multiple comparisons tests were performed using Graphpad Prism 6.0 to compare mean values between the groups. *P*-values < 0.05 were considered statistically significant.

## Additional file

Additional file 1:
**Interactions between Cx37 and Cx40 at cell-cell interfaces between mouse aortic endothelial cells.**

## Abbreviations

ACh: Acetylcholine
ANOVA: Analysis of variance
ATP: Adenosine triphosphate
cAMP: cyclic adenosine monophosphate
CRC: Concentration-response curve
Cx37: Connexin37
Cx40: Connexin40
DNAse: Deoxyribonuclease
eNOS: endothelial nitric oxide synthase
INDO: Indomethacin
KRB-buffer: Krebs-Ringers bicarbonate-buffered physiological salt buffer
L-NAME: *N*^ω^-nitro-l-arginine methyl ester
NO: Nitric oxide
PGI_2_: Prostacyclin
PHE: Phenylephrine
RNA: Ribonucleic acid
sGC: soluble guanylyl cyclase
SNP: Sodium-nitroprusside
vWf: von Willebrand factor
WT: Wild-type
