# Supplementary figures and images for: Endothelial Connexin37 and Connexin40 participate in basal but not agonist-induced NO release
Source: Cell Commun Signal. 2015 Jul 22;13:34. doi: 10.1186/s12964-015-0110-1 (PMC4510910; doi:10.1186/s12964-015-0110-1)

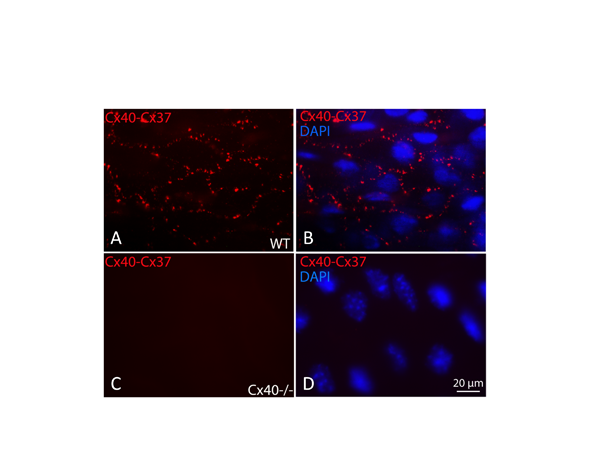

Supplement: Additional file 1: — Interactions between Cx37 and Cx40 at cell-cell interfaces between mouse aortic endothelial cells. [file 12964_2015_110_MOESM1_ESM.tif]
